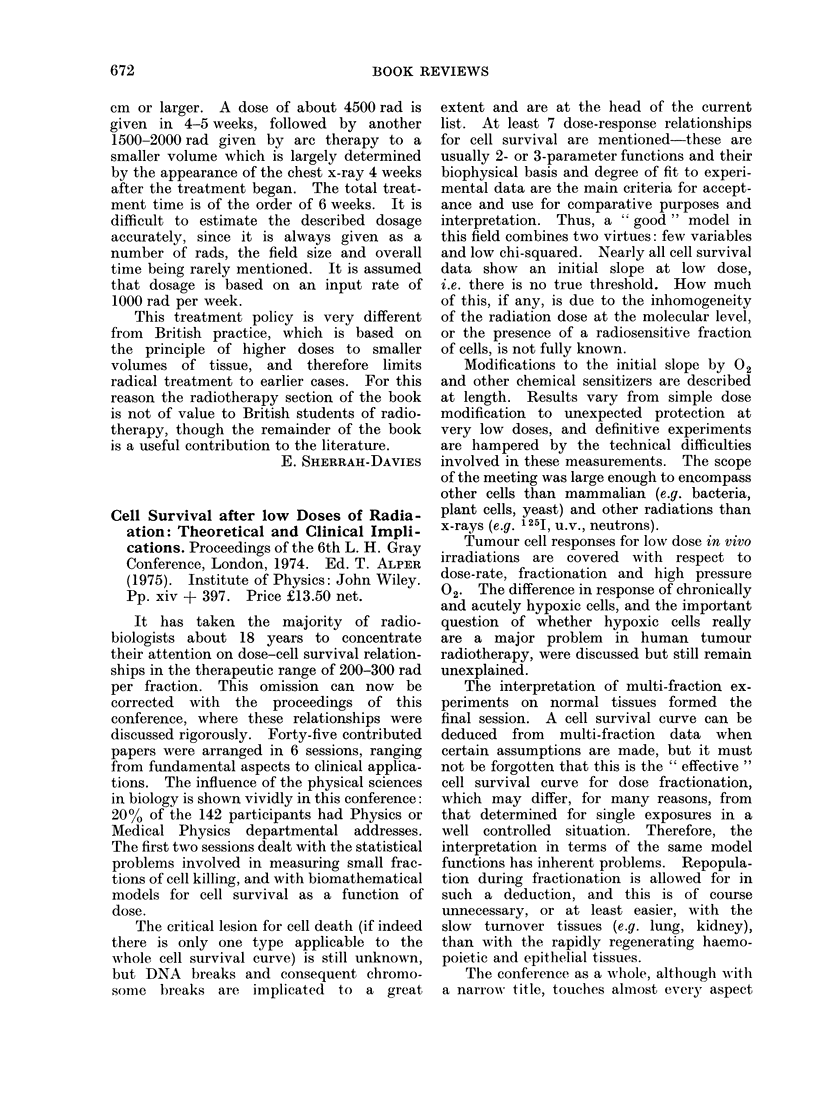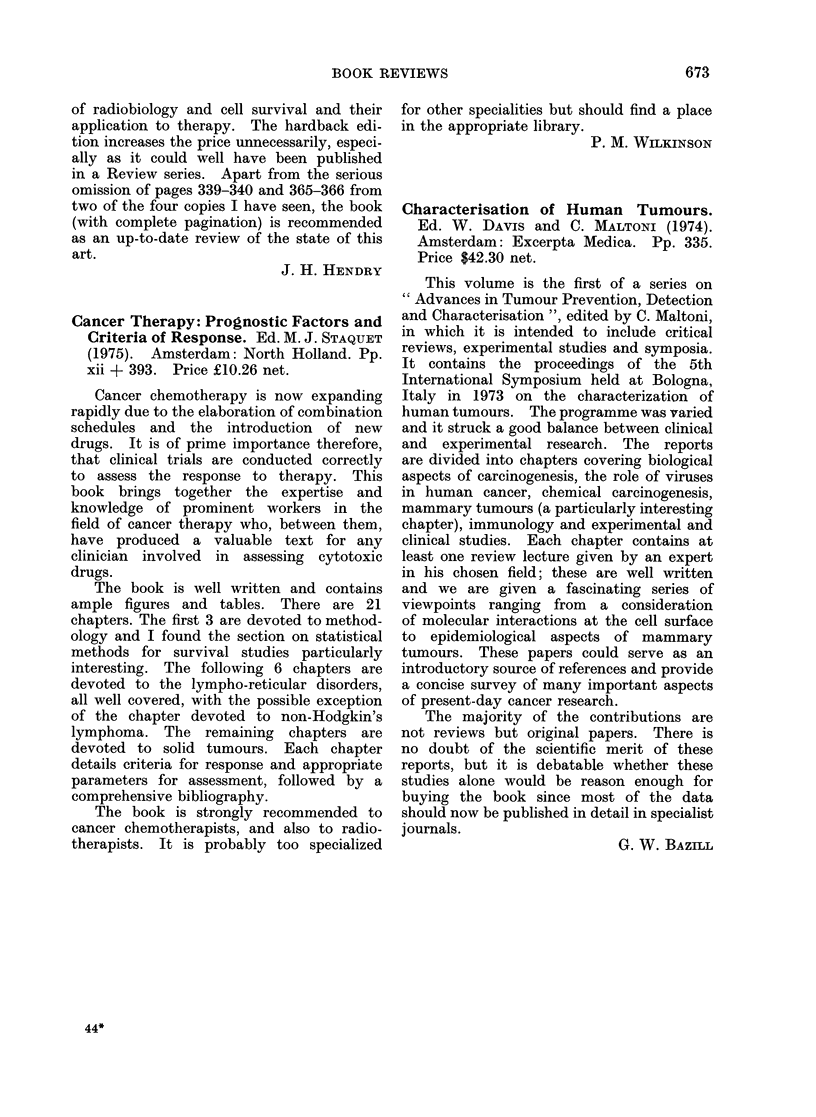# Cell Survival after low Doses of Radiaation: Theoretical and Clinical Implications

**Published:** 1976-06

**Authors:** J. H. Hendry


**Cell Survival after low Doses of Radiation: Theoretical and Clinical Implications.** Proceedings of the 6th L. H. Gray Conference, London, 1974. Ed. T. Alper (1975). Institute of Physics: John Wiley. Pp. xiv + 397. Price £13.50 net.

It has taken the majority of radiobiologists about 18 years to concentrate their attention on dose–cell survival relationships in the therapeutic range of 200–300 rad per fraction. This omission can now be corrected with the proceedings of this conference, where these relationships were discussed rigorously. Forty-five contributed papers were arranged in 6 sessions, ranging from fundamental aspects to clinical applications. The influence of the physical sciences in biology is shown vividly in this conference: 20% of the 142 participants had Physics or Medical Physics departmental addresses. The first two sessions dealt with the statistical problems involved in measuring small fractions of cell killing, and with biomathematical models for cell survival as a function of dose.

The critical lesion for cell death (if indeed there is only one type applicable to the whole cell survival curve) is still unknown, but DNA breaks and consequent chromosome breaks are implicated to a great extent and are at the head of the current list. At least 7 dose-response relationships for cell survival are mentioned—these are usually 2- or 3-parameter functions and their biophysical basis and degree of fit to experimental data are the main criteria for acceptance and use for comparative purposes and interpretation. Thus, a "good" model in this field combines two virtues: few variables and low chi-squared. Nearly all cell survival data show an initial slope at low dose, *i.e.* there is no true threshold. How much of this, if any, is due to the inhomogeneity of the radiation dose at the molecular level, or the presence of a radiosensitive fraction of cells, is not fully known.

Modifications to the initial slope by $O_2$ and other chemical sensitizers are described at length. Results vary from simple dose modification to unexpected protection at very low doses, and definitive experiments are hampered by the technical difficulties involved in these measurements. The scope of the meeting was large enough to encompass other cells than mammalian (*e.g.* bacteria, plant cells, yeast) and other radiations than x-rays (*e.g.* $^{125}I$, u.v., neutrons).

Tumour cell responses for low dose *in vivo* irradiations are covered with respect to dose-rate, fractionation and high pressure $O_2$. The difference in response of chronically and acutely hypoxic cells, and the important question of whether hypoxic cells really are a major problem in human tumour radiotherapy, were discussed but still remain unexplained.

The interpretation of multi-fraction experiments on normal tissues formed the final session. A cell survival curve can be deduced from multi-fraction data when certain assumptions are made, but it must not be forgotten that this is the "effective" cell survival curve for dose fractionation, which may differ, for many reasons, from that determined for single exposures in a well controlled situation. Therefore, the interpretation in terms of the same model functions has inherent problems. Repopulation during fractionation is allowed for in such a deduction, and this is of course unnecessary, or at least easier, with the slow turnover tissues (*e.g.* lung, kidney), than with the rapidly regenerating haemopoietic and epithelial tissues.

The conference as a whole, although with a narrow title, touches almost every aspect of radiobiology and cell survival and their application to therapy. The hardback edition increases the price unnecessarily, especially as it could well have been published in a Review series. Apart from the serious omission of pages 339–340 and 365–366 from two of the four copies I have seen, the book (with complete pagination) is recommended as an up-to-date review of the state of this art.

J. H. HENDRY